# Pharmacy Technicians, Stigma, and Compassion Fatigue: Front-Line Perspectives of Pharmacy and the US Opioid Epidemic

**DOI:** 10.3390/ijerph18126231

**Published:** 2021-06-09

**Authors:** Alina Cernasev, Shane Desselle, Kenneth C. Hohmeier, Joanne Canedo, Britney Tran, James Wheeler

**Affiliations:** 1 Department of Clinical Pharmacy and Translational Science, College of Pharmacy, The University of Tennessee Health Science Center, 301 S Perimeter Park Dr, Suite 220, Nashville, TN 3721, USA; khohmeie@uthsc.edu (K.C.H.); jms377@uthsc.edu (B.T.); jwheeler@uthsc.edu (J.W.); 2 Department of Social and Behavioral Pharmacy, Touro University California College of Pharmacy, 1310 Club Drive, Vallejo, CA 94592, USA; shane.desselle@tu.edu (S.D.); joanne.canedo@tu.edu (J.C.)

**Keywords:** stigma, opioid use disorder, pharmacy technician

## Abstract

The opioid epidemic in the United States has led to a quadrupling of opioid overdoses since the 1990s. Stigmas exist among healthcare professionals, and it is essential to educate the next generation of pharmacy technicians regarding opioid use disorder. The main objective of this study was to characterize the phenomenon of stigma through the pharmacy technician lens when taking care of patients who are using opioid medications. Grounded in Van Manen’s phenomenological approach and the Link and Phelan stigmatization model, a qualitative study was conducted from February to June 2020 to understand pharmacy technicians’ perceptions and attitudes towards patients using opioid medications. Focus groups (*n* = 46) with pharmacy technicians were conducted in-person and online over five months in 2020. Thematic analysis identified three themes that characterize the stigma and the relationship between pharmacy technicians and patients taking opioid medications: (1) pharmacy technician perspectives on stigma and patients with addictive opioid-use behavior; (2) current approaches of pharmacy technicians towards patients with addictive opioid-use behavior; (3) future approaches of pharmacy technicians towards patients with addictive opioid-use behavior. The findings highlight an “ever-present” negative connotation associated with the stigma that is formed from patient interaction. It is necessary to develop proper resources and educational materials to manage the stigma that exists in pharmacies throughout the nation. These resources will facilitate how to address and prevent the stigma among pharmacy technicians in the U.S.

## 1. Introduction

The United States (U.S.) has experienced an increase in the opioid crisis for decades, with overdose (OD) deaths quadrupling since the late 1990s, averaging 115 deaths per day [1]. In 2014, the U.S. experienced a total of 47,055 OD-related deaths [2]. These opioid-related deaths include those caused by both illicit and prescription opioids [3]. Further complicating the epidemic is the fact that the sources of these illicit opioids, such as heroin and carfentanil, are uncertain and therefore increase the risk of both fatal and nonfatal OD [4]. Between 2010 and 2015, system records of OD death rates in all 50 states found that deaths rates had increased in 30 states, including the District of Columbia, while in 19 states they were found to remain the same [5].

Foundational to the opioid crisis in the U.S. is the concept of stigmatization of those who have opioid use disorder (OUD) [6]. In an article concerning “Symbolic Interaction Stigma”, the authors discuss the impact stigma has on the stereotypes of an individual with mental illness [7]. However, “Symbolic Interaction Stigma” differs from the internalization for stereotypes because it considers the negative anticipatory thoughts individuals might have. “People seek to foretell what others might think, conjure notions about what could transpire, and imagine useful strategies to achieve desired ends—all before an interaction takes place”.

Link et al. distinguished four concepts of the “Domain of Symbolic Interaction” [7,8]. The first concept relates to perceptions of societal-level devaluation and discrimination, which discusses how stigmatized individuals might worry about others’ thoughts and, in turn, miss out on events and opportunities because they think it could lead to anticipated negative reactions. The second concept is stigma consciousness, which addresses race, gender, and sexual minority basis. “Stigma consciousness is an anticipation of being stereotyped and having the stereotyped status be a central feature guiding how others evaluate and relate to you” [7]. The third concept is rejection sensitivity, which refers to “the anxious expectation of rejection from others”. This concept states the example of an African-American participant imagining he is in the setting of a pharmacy and asks how concerned he might be that a clerk is glancing at him because of his race/ethnicity. Lastly, the fourth concept is anticipation of rejection. This concept goes beyond another’s actions or thoughts. “Anticipation of rejection focuses on the person’s own forecasting of whether rejection will occur” [7]. Because stigma can cause such an in-depth response to those living with mental illness or OUD, it is imperative for healthcare providers to understand these concepts above and beyond those of the biological mechanisms of addiction and dependence.

The public can be predisposed to these three components of stigma due to lack of understanding and education [9]. This lack of understanding can drive individuals to become more closed off about their OUD, leading to a barrier to treatment [10]. Addiction disorder is problematic enough for patients, and challenges with accessing and acquiring needed treatment are further exacerbated by prevailing stigma, which further discourages individuals who might otherwise seek help [11]. Unfortunately, healthcare providers are not precluded from experiencing stigma against patients with OUD. Using stigmatized terms such as “substance abuser” can also lead healthcare providers to provide suboptimal care [12]. With an inability to turn even toward licensed health professionals, patients with OUD may feel isolated and as if no one is there to support them [13].

The National Academies of Sciences, Engineering, and Medicine (NAEM) state that stigma can impede individuals with mental illness (MI) or substance use disorder (SUD) from partaking in beneficiary prevention programs [14]. Individuals with SUD will go to some lengths to avoid the ridicule that comes with stigma. NAS also reported that there is significantly less research conducted regarding SUD than MI. When comparing the two, NAS examined 1500 peer reviewed papers for MI and only 200 for SUD. With an escalation of the country’s opioid crisis, the organization averred the need for more research regarding OUD-related stigma and its impact.

Among healthcare providers, pharmacists and pharmacy technicians often serve on the frontline of care with many patients, including those with OUD. They are widely accessible (typically not requiring an appointment) and are frequently seen by patients, refilling their prescriptions and purchasing over-the-counter medicines. Pharmacy technicians often serve as the point of entry into the pharmacy, if not the entire health care system [15]. They have been referred to as the “face” of community pharmacy, serving as the primary liaison and point of communication between the patient and the pharmacy team. Furthermore, a Massachusetts study concerning pharmacy technicians was designed to assess the attitudes towards dispensing and administering naloxone. This study took place in retail pharmacies across the state, including high-risk municipalities (HRMs) and low-risk municipalities (LRMs) [16]. Results found that the pharmacy technicians from both HRMs and LRMs were comfortable and willing to assess the need for and administration of Naloxone to patients.

Thus, the objectives of this study were to characterize the phenomenon of stigma through the pharmacy technician lens when taking care of patients who are using opioid medications.

## 2. Materials and Methods

### 2.1. Study Settings and Sampling

Approval for this study was granted by the Institutional Review Board (IRB). Verbal informed consent was obtained prior to each focus group, in which the procedure was explained to participants and any questions about taking part were answered. Participants for this study were obtained via the primary institution’s Continuing Education email listserv. Participants self-selected their participation, and inclusion criteria were (1) adults, (2) active pharmacy technicians working in the community/retail pharmacy setting, (3) English speakers, (4) familiarity with the topic and (5) willingness to discuss their opinion. Participants received a $50 gift card as compensation for their participation.

### 2.2. Data Collection

The Focus Group Discussions (FGD) guide was developed based on Stigma Conceptualization [8] and Phenomenology [17]. The questions aimed to examine participant perceptions and experiences regarding stigma, among other aspects of pharmacy technician–patient communication. The FGD was comprised of open questions addressing views on stigma associated with OUD. From March until June 2020, FGD took place in-person and online to accommodate the COVID-19 pandemic [18]. One researcher with qualitative research training and stigma expertise led the FGD.

Before the FGD, the following background information was collected from participants: state of licensure, number of years in practice, type of pharmacy practice, and gender. All FGD were audio recorded before being transcribed verbatim. The FGD emphasized that the participants were the experts on the topic and used open ended questions to encourage participants to speak about their experiences.

### 2.3. Data Analysis

Thematic analysis involving an inductive approach was used to identify themes and subthemes [19]. Two researchers with qualitative methods expertise performed a line-by-line reading of the first two FGD transcripts, after familiarizing themselves with the corpus of data. The researchers met to define the initial codes and identify emergent categories, which were grouped into themes.

A process of independent review and open discussion was used to clarify and revise the coding frame until consensus was reached [20]. The coding process was facilitated by a qualitative software, Dedoose^®^ (v2.0, Manhattan Beach, CA, USA), which was used for generating initial codes and developing and reviewing themes. Two researchers reviewed the final codes, and one researcher identified common and recurrent themes and subthemes [20].

The researchers used Yardley’s criteria to ensure that the quality and rigor of qualitative research were met [21]. For example, sensitivity to context criteria was obtained by reviewing the previous literature when writing the FGD open ended questions. Furthermore, the team endeavored to recruit participants from several states to gain a broader understanding of this stigma phenomenon. For commitment and rigor criteria, all stages outlined by Braun and Clarke were followed [19]. Furthermore, the researchers followed the consolidated criteria for reporting qualitative research (COREQ) throughout the study design and data analysis [22]. The credibility criterion was achieved by having two researchers checking the identified codes and the emergent themes. Furthermore, reflective memos were written by the researchers throughout the process of data collection and analysis [23]. All the authors involved in the data collection and analysis disclosed their positions regarding the research topic through a memo [23].

## 3. Results

A total of eight focus groups were conducted between March and June 2020 and, 46 participants attended. On average, the focus groups lasted 56 min. The first focus group was in-person, and the other seven focus groups were conducted online due to the ongoing pandemic. Out of 46 participants, 10 were male and the rest identified as female. The majority of the participants were from two states: Tennessee and California. Other participants were working in the following states: New Jersey, Florida, Georgia, and Alabama. Out of 46 participants, one was not a certified pharmacy technician.

Thematic analysis revealed three themes, which illuminate pharmacy technicians’ perspectives on OUD-related stigma when interacting with patients. The first theme identifies the participants’ definition of and views on the stigma associated with opioid use among patients. The second theme explores the current state of interactions between pharmacy technicians and patients with opioid related medications. Finally, the third theme explores the perspectives of technicians on future means to improve care and reduce stigma for patients with OUD.

### 3.1. Theme 1: Pharmacy Technician Perspectives on Stigma and Patients with Addictive Opioid-Use Behavior

Participants associated a negative approach and reaction with the stigma that is formed towards opioid using patients. The participants shared their opinions about the definition of the term ‘stigma’ and their experiences that had influenced their assumptions. Participants had similar definitions in that stigma leads to more of “… a negative perception…” in the pharmacy (P7).

P11 perceives stigma as a burden for patients from which they might not escape. She describes the stigma as a process in which both the patient and the pharmacy technician participate together. However, it is the stigma associated with opioid medications that controls the patients’ lives.

“There is a stigma with patients…it’s kind of like with being prejudice…or… profiling a patient like how they come to you and how they approach you when they drop off their prescription or if it’s electronically sent and if they’re picking it up. How they are defensive against why am I asking for their ID or you know any other questions like if I’m asking their date of birth. It’s all defense mechanisms on them.”

(P11)

P18 vividly describes his opinion on the origin of stigma and how views the patient as being trapped in a vicious life cycle:

“… regarding the stigma for taking you know Norcos^®^… I think…as a technician it’s the first impression you give to the patient. Whenever they do come in it doesn’t matter if they’re addicted or not just treat them as a normal person… Get them educated where they know if they actually do need the medication and they have enough courage to actually go to the doctor and get the medication and not suffer.”

(P18)

Several of the participants suggested that talking and behaving professionally with the patient is an important step. Furthermore, P22 states that being ‘front liners” brings more responsibility in developing a professional and non-judgmental relationship.

“… I feel like so over the years, [we] are on the front line dealing with people that get these types of drugs. You, you don’t mean to be automatically think they have a drug problem just by looking at their hard copy or their prescription you automatically think over they’re a drug addict or have a drug a drug problem, but that’s not always the case. You always have to be so open minded to realize it’s not always the case if their appearance is a problem…”

These perceptions were influenced by negative interactions between pharmacy staff and patients displaying signs of addictive opioid-use behavior as well as opinions formed outside of the pharmacy itself. These experiences can be further broken down into two subthemes: *aggressive encounters between opioid use patients and health professionals*; and *public portrayals of opioid-use patients*.

#### 3.1.1. Sub-Theme: Aggressive Encounters between Opioid Use Patients and Health Professionals

Participants describe opioid use patients as easily agitated and demanding if they cannot get their opioid prescription filled right away. P7 describes these patients:

“They get angry easily… they are very demanding… they’re usually the ones who are staying late. Like… our pharmacy is supposed to close at 6PM and they’re the ones who show up at 6:30 or, or call us like at 5:55 and want us to stay later for them like on a Friday night or different things like they are some of the most problematic patients I’ve had.”

(P7)

Besides these urgent demands to get their prescriptions filled regardless of whether the refill is too early, participants also describe the patients they interact with as very impatient:

“Ready to leave cause they-I think they’ve been there all day for their appointments and you know… sometimes they just get very impatient. So, they just get real agitated and walk out, come back, and walk out, come back and yeah it’s just they can’t sit still. It’s just, it’s a lot and they sit there and stare at you and I’m like okay but we’re still working on it, getting it done for you.”

(P4)

#### 3.1.2. Sub-Theme: Public Portrayals of Opioid-Use Patients

Participants shared their perspectives on the reasons underlying the negative behaviors associated with the development of the opioid stigma. These participants explain the tension that is sensed in patients who come to pick up their opioid prescriptions in terms of “rejection sensitivity”. P11 states:

“… they’ve acknowledged that maybe they have an issue with it. And it’s not a continued use, it’s that they’re actually trying to get help. But I think a lot of those people are just really treated badly and they’re, they’re just embarrassed to pick up their medication at all, which, I don’t think that should be the case. Because I think it is a problem. I think it is overprescribed to some extent, but it does seem that there’s kind of a stigma with people trying to get treatment for that also.”

“Stigma consciousness” was also discussed as self-consciousness among opioid use patients, with participants further speculating that social media plays a big part in the negative stigma concerning opioids and addiction, and the patients who use them:

“I also think that the media will somewhat have a play in it… We always hear things about how a certain state has this many people that are overdosing, or this many opiates that have been dispensed by state and when someone sits down and listens to that kind of stuff, they might think like, ‘oh,’ you know, ‘it’s, it’s really a problem.’ But, I feel like again they don’t understand that not everybody is addicted to this drug, or they have to have it, you know, that thing.”

(P14)

### 3.2. Theme 2: Current Approaches of Pharmacy Technicians towards Patients with Addictive Opioid-Use Behavior

Participants mention various issues regarding opioid medications and patients. Based on their interactions, participants best describe their experiences that can be divided into the following sub-themes.

#### 3.2.1. Sub-Theme: Interactions between Opioid-Use Patients and Technicians

Participants share their perspectives on their reactions and the ideal behaviors that they consider necessary to approaching opioid-use patients in a professional manner.

Pharmacy technicians described their current experiences with patients with signs of addictive opioid-use.

“…We have to call the doctor sometimes and sometimes they just get very impatient. So, they just get real agitated and walk out, come back, and walk out, come back and yeah it’s just they can’t sit still. It’s just, it’s a lot and they sit there and stare at you and I’m like okay but we’re still working on it, getting it done for you.”

(P4)

Pharmacy technicians often referred to “early refills”, a term that describes a situation where a patient with an opioid prescription comes into the pharmacy for an opioid fill even though the patient should still have opioid medication on hand based on a prior fill. “Early fills” were viewed as a sign of possible addictive opioid-use. The situation surrounding an “early refill” can affect the interaction between technician and patient. The following examples describe these types of situation.

“So usually you see a pattern when they have these behavior or signs and so therefore… they always asking for early refills um you know not having. Refill their medication always early…repetitively…calling yelling screaming…”

(P1)

The “early fill” is a relative designation made by either a third-party payer (i.e., medical insurer, Medicaid, Medicare), physician, or pharmacist. Participants noted that these vary, though the stricter of the dates is the one followed. Pharmacy technicians viewed the requesting of a specific brand or manufacturer of an opioid as unusual, as compared to other non-opioid medications, and as being similar to “early fills” as a sign of addictive opioid-use.

“… the barrier that we struggle with a lot is dates… you can only do two days early (at our pharmacy), you can only do three days early or—you know then we have some doctors who only ask to fill every thirty days and umm then with another barrier is patients are brand specific and some brands are hard to get or they’re really expensive and it cost more and more get stuck with the over.”

(P35)

Lastly, pharmacy technicians described overcommunication around an opioid prescription fill as a sign of addictive opioid-use.

“… Umm just constantly trying to fill their prescription early, umm always coming up with them and the elaborate story of why they need to fill their prescription early…”

(P20)

#### 3.2.2. Sub-Theme: Current Approach to Negative Opioid-Use Patient Interactions

Participants express their thick skin when it comes to handling negative interactions with these patients:

“You have to just do your job. You know? But…you do have to have for lack of a different term maybe bedside manners when it comes to being a pharmacy technician… no you’re not gonna get it, I’m sorry you’re two refills you were in here yesterday. You can’t be that way. I mean there are some technicians that have that finesse and some people that don’t. It’s just a matter of personality I would say but I mean I think that everybody should have some sort of empathy or should try and then—”

(P39)

Other participants also emphasize the importance of compassion and to be judgment free when it comes to their approach to these patients:

“… we should not judge a patient ‘cause no matter how the patient have the pain or if it’s an addiction it’s still a process that they have to go through… we should not judge them on how they take their medication…”

(P1)

#### 3.2.3. Sub-Theme: Effects of Negative Opioid-Use Interactions on Quality of Work Life

Participants describe how patients’ interactions with them affect their work environment as well as their overall well-being at work. P 29 feels the impact of the interactions through the social media’s lens:

“I also think that the media will somewhat have a play in it… when someone sits down and listens to that kind of stuff, they might think like, ‘oh,’ you know, ‘it’s, it’s really a problem.’ But, I feel like again they don’t understand that not everybody is addicted to this drug…”

(P29)

### 3.3. Theme 3: Future Approaches of Pharmacy Technicians towards Patients with Addictive Opioid-Use Behavior

Pharmacy technicians contrasted current practice with ideal practice. There was an understanding that the status quo was inadequate to address the growing need to provide care to patients with signs of addictive opioid-use. Participants made recommendations and suggested best practices to address observed problems in opioid-use care. Subthemes included the right approach to patient care and the responsibilities of the pharmacy technician.

#### Sub-Theme: The “Right” Patient Care Approach

Pharmacy technicians suggested that a compassionate approach to patients may overcome stigma. Participants further explained an ideal approach to these patients is one where “…you have to have some sort of compassion and think of the way that a patient feels as far as stigmatizing them.” (P4)

They felt that awareness of implicit biases toward patients filling opioids, especially those with suspected addictive opioid filling behaviors, was warranted:

“… not forming an opinion… yeah and not being reactionary is another part. Trying to understand where they’re coming from, cause sometimes opioid use, the stigma that comes with it is that they were just drug addicts to begin with…and trying to understand where they are coming from building up repertoire with them. And giving them the benefit of the doubt more often than not because I’m not the doctor, I don’t know what the best treatment for that person is.”

(P29)

“… I would start with telling the, the technician to, don’t form an opinion right off the bat. Um whether it be a prescription for Amoxicillin or Norco, the patient probably needs the medication for specific reason and so we don’t need to be judging them from the get go um we need to give them the benefit of the doubt. try to educate yourself as well on the symptoms and signs and how opiate-opioid addiction affects people’s like mental status so you get more of a understanding and more sympathetic towards their situation instead of getting angry when they get angry at you. Which is very hard, but I think it’s better if you understand where their coming from.”

(P28)

### 3.4. Responsibilities of Pharmacy Technicians in Optimizing Patient Care

Lastly, the participants describe what they feel their responsibilities are when it comes to optimizing care for opioid-use patients, and some also suggest that technicians should contribute in counseling patients on opioid medications. Participants feel that an important role of pharmacy technicians is to be an active listener for patients:

“Asking questions interactively open ended uh open ended questions to get an answer from the patient to see why they’re getting it and then if so, you can help determine to the pharmacist like maybe we should contact the doctor of if there is a therapy change what if you know like should the doctor be prescribing more or less…”

(P11)

Technicians also indicated that continuing education and professional development around opioid use and opioid use disorders is necessary to improve that current state of practice.

“I think also… really educating yourself. A lot of times, I think some technicians don’t feel empowered to educate themselves. You do have a license also, so educating yourself and knowing what you’re able and not able to do and somehow catching those things in the front end really kind of… reduces, you know, how angry the patient will be…”

(P41)

## 4. Discussion

This study sought to explore prevailing attitudes and encounters between patients with new or refilled prescriptions for opioid products and pharmacy technicians, who are on the front line of care and among the health professionals most frequently seen by patients with OUD or patients who receive any prescription for an opioid medication. Data revealed a complex array of feelings and interactions described by technicians, displaying compassion and empathy for patients with pain, such as those suffering from terminal illness, but also frustration in dealing with patients who seek early refills or who might be suspected to be attempting to “game the system” by acquiring prescriptions from multiple prescribers or attempting to obtain extra doses of their medication. In spite of compassion for suffering patients in a broader sense, technician study participants expressed their wariness of individual patients receiving opioids, feeling that patients often attempt to be secretive in providing medication histories and become easily agitated in the course of routine pharmacy care. Pharmacy technicians believe that they have developed or need to develop a thick skin to deal with these patients. This phenomenon is not unique to pharmacy technicians, and has been referred to “compassion fatigue” in the literature and is present across the health professions [24,25]. In fact, compassion fatigue has been identified through qualitative research methods among first responders in the opioid epidemic [26,27,28]. Our research suggests a similar phenomenon is present here among the front-line pharmacy workforce in the United States.

Healthcare providers need to be empathetic towards people with OUD [29]. They need to be mindful of their word choices and avoid any labeling that could be interpreted as negative. Healthcare providers who are able to understand the hardships of individuals with OUD will be able to give them the proper attention and care needed [30]. With their support, patients with OUD will experience an improved quality of life. This support will lead to an increase in individuals with OUD feeling comfortable in seeking and remaining on treatment and gaining access to lifesaving therapies, such as naloxone, decreasing the number of opioid related deaths in the future [31].

While developing a proverbial thick skin may not a bad idea for any health professional, particularly those dealing with the public en masse, as is the case with pharmacy technicians, a more proactive and salubrious approach would be to regard all patients as requiring expert and sensitive care and forgoing any attitude that certain patients or types of patients are “problem patients. Thinking of patients in such a negative manner can compromise the quality of care [32]. Once a patient has been labeled in this way, it is challenging for the health professional to reverse his or her opinion and provide the most optimal care [33]. It has been suggested that careful, reflective practice that involves further divesting of ego in the practitioner will mitigate these occurrences, improve care, and even result in a more positive work environment [34].

Officers from the National Institute on Drug Abuse (NIDA) recommended a very substantial role to be played by pharmacists in stemming the tide of prescription drug abuse [35]. In doing so, they urged pharmacists and staff (technicians) to acquire accurate medication histories, whose success will hinge upon completeness, accuracy, and effective communication devoid of attitudes that hinder optimal care. These officers also stressed the importance of prescription drug monitoring programs (PDMPs). It has been suggested that technicians and other support personnel not only assist with administrative components of PDMPs but also help with fostering positive provider–patient relationships that ultimately render these PDMPs more effective [36].

It has been observed that having contact with patients with mental illness reduces stigma for pharmacy students [37]. While not guaranteed, it could be postulated that similar contact with patients using opioids could reduce stigma in technician education. Incorporation of such experiences as well as inclusion of greater didactic and experiential training in empathy, communication skills, and ethical judgment has been recommended [38]. However, given the lack of standardization in technician vocational and educational requirements across institutions and across states [39], more immediate strides will have to be accomplished through employer on-the-job training, accompanied perhaps by simulation training accompanying technician certification [40].

Olsen and Sharfstein (2014) suggest that the prevalence of stigma is heightened further by the deaths of well-known celebrity figures at the hand of overdosing [41]. They add that while there is regret and sadness over these deaths, there is also an opportunity, taken by many, to further look upon substance abuse as simply a matter of moral weakness and poor choices by irresponsible people who do not care enough for the people who love and even idolize them. They frame the mitigation of stigma as a matter of language; that is, health care practitioners should not only have respectful attitudes, but adopt accurate, nonjudgmental language to describe this disorder and those whom it affects. The public can fight back against the rising threat of overdose by supporting broad access to effective treatment with medications. The notion of role-modeling proper language and behaviors to peers was corroborated in a study of practitioner reactions to various vignettes reported by Goodyear et al. [12].

A recent scoping review of opioid-related stigma indicated that its prevalence depends upon the type of opioid being consumed and the social identity and networks of the person consuming it [42]. The review found that stigma permeates intrapersonal, interpersonal, structural, and societal levels, with people who consume opioids being marginalized at all levels. The current study sheds additional light on this matter, as opioid users are somewhat pigeon-holed by the pharmacy technicians interviewed. However, as recommended by McCradden et al., interventions and strategies to mitigate stigma must be multifaceted [42]. Commensurate with their findings and recommendations, results from the current study would suggest that pharmacy technicians be educated to consider OUD as a disease that must be treated, and that patients with any type of condition might seemingly operate on a “short fuse” given the stress of having such a condition and given what might be a lack of social support by family and peers. Technicians would also benefit from training to have them eschew media and other lay portrayals of opioid users and patients with any substance abuse disorder.

Studies of organizational culture would suggest that beyond education and training, an ethos for providing safe and effective care permeates throughout an employer’s personnel. Indeed, a study examining stigma reported that messages both top-down and across provider peers in an organization, particularly when supported and codified by its core values, are instrumental in mitigating stigma and its effects on care [43]. In the current study, technicians expressed empathy for patients’ conditions necessitating their use of potent drugs. They also expressed a feeling of responsibility for providing high-quality care even in spite of some stigma-related attitudes. As such, strengthening culture and role modeling behaviors would appear to be feasible and have an indelibly positive effect on pharmacy technicians who only want to do the best they can for their patients.

### Strengths and Limitations

This is the first study to explore pharmacy technicians’ perceptions of stigma and opioid use disorder in the U.S. The perceptions and biases unearthed provide innovative Continuing Education opportunities to highlight and address these issues in order to normalize interactions with patients impacted by OUD.

The sample was comprised of participants located in geographically diverse states where the laws and regulations vary, and some states have been burdened by OUD and the stigma associated with it. Recruitment was conducted from a large continuing education email listserv, and technicians self-selected for participation. Because of the authors’ affiliations with two states, an over-representation of individuals from those two states occurred in our FGD sample. These factors could have contributed to selection biases. Furthermore, we made considerations throughout the study to enhance the validity and trustworthiness of the findings.

## 5. Conclusions

This study’s data presented in-depth general perceptions of pharmacy technicians on the stigma associated with prescription opioid use. The findings highlight an “ever-present” negative connotation associated with the stigma that is formed from patient interaction. This study pointed out the origins of the stigma, the factors that could diminish the stigma, and the importance of compassion and sympathy during interactions with patients. When interacting with patients with OUD, one of the recommendations that emerged from the data was not to show any judgment, while other suggestions were to keep calm and not be argumentative.

Further, proper resources and educational materials are necessary, to be developed, implemented, and evaluated nationwide. These resources will facilitate how to address and prevent stigma among pharmacy technicians in the U.S.

## Data Availability

To respect the confidentiality of the participants and data, this study did not report any collected data.
